# Virulence Genes and Antimicrobial Resistance Profiles of *Pasteurella multocida* Strains Isolated from Rabbits in Brazil

**DOI:** 10.1100/2012/685028

**Published:** 2012-07-31

**Authors:** Thais Sebastiana Porfida Ferreira, Maria Roberta Felizardo, Débora Dirani Sena de Gobbi, Cleise Ribeiro Gomes, Pedro Henrique de Lima Nogueira Filsner, Marina Moreno, Renata Paixão, Jucélia de Jesus Pereira, Andrea Micke Moreno

**Affiliations:** ^1^Programa de Epidemiologia Experimental Aplicada às Zoonoses, Laboratório de Sanidade Suína e Virologia, Faculdade de Medicina Veterinária e Zootecnia, Universidade de São Paulo, São Paulo, SP, Brazil; ^2^Laboratório de Sanidade Suína e Virologia, VPS/FMVZ/USP, Cidade Universitária, Avenida Prof. Dr. Orlando Marques de Paiva 87, CEP 05508 270 São Paulo, SP, Brazil

## Abstract

*Pasteurella multocida* is responsible for a wide range of diseases in domestic animals. In rabbits, the agent is related to nasal discharge, pneumonia, otitis media, pyometra, orchitis, abscess, and septicemia. One hundred and forty rabbits with respiratory diseases from four rabbitries in São Paulo State, Brazil were evaluated for the detection of *P. multocida* in their nasal cavities. A total of twenty-nine animals were positive to *P. multocida* isolation, and 46 strains were selected and characterized by means of biochemical tests and PCR. *P. multocida* strains were tested for capsular type, virulence genes, and resistance profile. A total of 45.6% (21/46) of isolates belonged to capsular type A, and 54.34% (25/46) of the isolates were untypeable. None of the strains harboured *toxA* or *pfhA* genes. The frequency of the other twenty genes tested was variable, and the data generated was used to build a dendrogram, showing the relatedness of strains, which were clustered according to origin. Resistance revealed to be more common against sulfonamides and cotrimoxazole, followed by erythromycin, penicillin, and amoxicillin.

## 1. Introduction

*Pasteurella multocida* is an important pathogen that infects a wide range of animal hosts, and it is part of the respiratory tract microbiota of different animal species [1]. In rabbits, infections by *P. multocida *are considered one of the most frequent diseases affecting the species. Pasteurellosis in these animals may present itself as rhinitis with purulent nasal discharge, pneumonia, otitis media, pyometra, orchitis, abscesses, and septicemia [2].

In the United States, more than 50% of the adult rabbits used in the industry are killed or removed from production due to infection by *P. multocida, *which represents a major economic loss [3]. Currently, control measures of pasteurellosis in rabbits involve treatment with antibiotics and the slaughter of infected animals. However, the treatment of infected animals only alleviates clinical signs and/or slows the progression of the disease, but it does not eradicate their infection [3].

Brazilian rabbitry production is mainly oriented to meat, skin, pelage, carcass, and blood, totalizing around 230,000 animals in 2010; however, the importance of rabbits as pet animals is growing in Brazil as well as in other countries [4]. With this in mind, it is also important to evaluate the zoonotic potential of *P. multocida* strains, when these animals have contact with children or immunosuppressed individuals [5].

Two reports of human infection by *P. multocida* after rabbit lick or byte were recorded in the last ten years. The first describes a case of meningitis and epidural, subdural, and subgaleal empyema in a 15-year-old boy. The second case reports an endovascular stentgraft infection caused by *P. multocida* after a bite by the patient's household rabbit, in a 68-year-old man [5, 6].

Data on the antibiotic resistance profile and virulence factors of the agent may lead to a better understanding of pasteurellosis epidemiology in rabbits. Reports describing the characterization of *P. multocida* isolated from rabbits in Brazil date back to the 1980s [7, 8]. Even though the molecular basis of the pathogenicity and host specificity of *P. multocida* is not well understood, several studies have reported that a number of virulence factors can be correlated with pathogenic mechanisms, and these factors have not yet been described in Brazilian strains [9].

The aim of this study was to evaluate the occurrence of *P. multocida* in rabbits, their resistance profile, as well as to investigate the presence of virulence genes coding for outer membrane and porin proteins (*oma87, ompH, plpB, psl*), adesins (*ptfA, fimA, hsf-1, hsf-2, pfhA, tadD*), neuraminidases (*nanB, nanH*), iron acquisition related factors (*exBD, tonB, fur, tbpA, hgbA, hgbB*), superoxidee dismutases (*sodA*, *sodC*), dermonecrotoxin (*toxA*), and hyaluronidase (*pmHAS*).

## 2. Material and Methods

### 2.1. Sample Collection and Processing

The samples were collected with sterile swabs from external nares of one hundred and forty rabbits with respiratory disease from four rabbitries in São Paulo State, Brazil. The swabs were placed in Amies transport medium (Copan Diagnostics Inc., California/USA) and kept under refrigeration until analysis.

Each swab was plated on tryptic soy yeast extract agar (Difco-BBL), supplemented with 5% of defibrinated sheep blood, and they were identified as *P. multocida* using standard biochemical procedures, including catalase, oxidase, indol production, urease activity, ornithine decarboxylase production, and carbohydrate fermentation [10] associated with PCR, for the detection of *kmt *species specific gene fragment [11].

### 2.2. Antimicrobial Susceptibility Testing

The determination of the susceptibility profile was performed with disc diffusion test dilution technique according to the standardized protocol of the M31-A3 Document, issued by the Clinical and Laboratory Standards Institute (CLSI) (CLSI, 2008). The antimicrobial agents tested included ceftiofur, penicillin, amoxacillin, florfenicol, norfloxacin, enrofloxacin, ciprofloxacin, tetracycline, doxycycline, sulfamethazine, trimethoprim-sulphamethoxazole, and erythromycin (Oxoid Ltd. Cambridge, UK). Reference strains *Escherichia coli* ATCC 25922 and *Staphylococcus aureus* ATCC 29213 were used as quality control organisms in all antimicrobial susceptibility tests.

### 2.3. DNA Preparation

The bacteria were cultured overnight in brain hearth infusion (BHI) broth at 37°C, and 200 *μ*L of this cell suspension was submitted to the DNA extraction procedure described by Boom et al. [12].

### 2.4. PCR Analyses and Gel Electrophoresis

*P. multocida* strains were evaluated for the presence of capsule biosynthesis genes *cap A, B, D, E,* and *F* [11] and the following virulence related genes: *oma87, ompH, plpB, psl, ptfA, fimA, hsf-1, hsf-2, pfhA, tadD, nanB, nanH, exBD/tonB, fur, pmHAS, tbpA, hgbA, hgbB, sodA, sodC, toxA,* and *pmHAS* [9, 13]. The following *P. multocida* strains were used as positive control: ATCC 12945, ATCC 12948, and NCTC 10323.

For all reactions, 5 *μ*L of DNA template was added to the 45 *μ*L mixture containing 20 pmoles of each primer pair (Invitrogen Corporation, California/USA), 1.5 mM of MgCl_2_, 200 mM of each dNTP, 1U of Taq DNA polymerase (Fermentas Inc. Maryland, USA), 1X PCR buffer, and ultrapure water. PCR conditions were carried out according to the respective authors' protocols. The amplified products were subjected to electrophoresis in 1.5% agarose gel, stained with BlueGreen (LGC Biotecnologia, São Paulo, Brazil), and identified by means of a 100 bp DNA ladder.

### 2.5. Statistical Analysis

Relatedness among *P. multocida* strains was determined by a comprehensive pairwise comparison of different genes combinations, using the Dice coefficient by means of the Bionumerics 6.6 software (Applied Maths NV, Sint-Martens-Latem, Belgium) to generate the dendrogram. The discriminatory index was calculated according to the method described by Hunter and Gaston [14].

## 3. Results

One hundred and forty animals were examined and twenty-nine were positive to *P. multocida* isolation (Table 1). From positive animals, 46 strains were characterized as *P. multocida* through biochemical tests and PCR. All 46 strains were tested for the five capsular types (A to F), and 45.6% (21/46) of isolates belonged to capsular type A and 54.34% (25/46) isolates were untypeable using the PCR described.

Strains were screened for the presence/absence of a total of twenty-two different genes coding for virulence factors, and the complete results are given in Table 2. Among the 46 *P. multocida* strains, the 22 virulence genes ranged in prevalence from 0% (*toxA, pfhA*) to 97.8% (*SodC*) (Table 2). Most of the strains presented the *ptfA* gene (93.4%), coding for type 4 fimbrial subunit, as well as putative nonspecific tight adherence protein D encoding gene* tadD *(91.3%). The gene locus *exbBD- tonB, *which is involved in energy-coupled transport of transferrin binding proteins through bacterial membrane spaces, was present in 60.8% of the *P. multocida* strains tested.

With respect to the genes encoding hemoglobin-binding proteins, the most frequent was* hgbA* (73.9%), followed by the *hgbB* gene (30.4%). Among genes encoding outer membrane proteins, *OmpH* (76%) and *Oma87* (84.7%) genes were more frequent than *OmpA* (28.2%) and *plpB* (34. 7%) genes. The* tbpA* (8.6%) and* fur *(4.3%) genes presented frequencies below ten percent.

**Figure 1:**
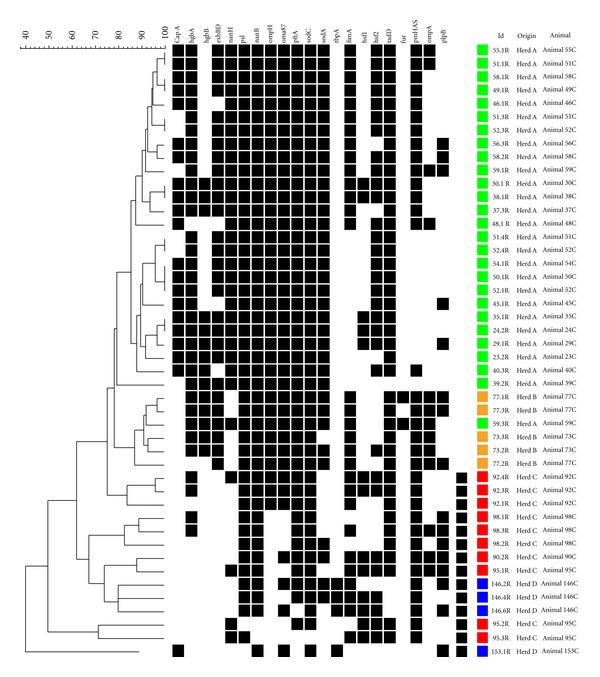
Dendrogram representing relatedness among *P. multocida* strains isolated from rabbits according to virulence profile.

By using the virulence genes profile, it was possible to build a dendrogram (Figure 1) that distributes the strains in 38 different combinations. Considering groups with more than 80% of similarity (presence or absence of up to 4 genes), it was possible to observe that strains from rabbitries A are clustered together, and with exception of a few cases, strains from the same animal are also clustered together. The discriminatory index of the virulence gene profile was 0.99.

Considering the resistance profile, 47.8% (22/46) of the strains were resistant to at least one of the tested drugs. The resistance was more common against sulfonamides and cotrimoxazole with 28.3% of strains (13/46), followed by penicillin with 10.9% (5/46), amoxicillin with 6.5% (3/46), and erythromycin with 4.3% (2/46). All tested strains were sensitive to ceftiofur, florfenicol, norfloxacin, enrofloxacin, ciprofloxacin, tetracycline, and doxycycline.

## 4. Discussion

Stahel et al. [15] describe a frequency ranging from seven to nearly 100% of *P. multocida* in the upper respiratory tract of rabbits of different origins. At present, the frequency observed was 20.7% (29/140), varying from 7.4 to 52.5% in four Brazilian rabbitries. Using PCR, less than 50% of the strains were identified as capsular type A, which is the capsular type most often described for rabbits [13]. The high frequency of untypeable strains for the gene encoding the capsule (54.3%) using PCR was an unexpected data.

Arumugam et al. [16] compared identification of capsular type in *P. multocida* from different hosts using conventional phenotypic methods as well as the PCR multiplex described by Townsend et al. [11]. The authors reported 19.3% (22/114) of strains untypeable using PCR, against 48.2% (55/114) of untypeable strains using the hyaluronidase test, and acriflavine flocculation for serogroup A and D identification, respectively, as well as serological test to capsular type B.

The negative results of all samples for gene *toxA *may be related to the fact that the gene encoding dermonecrotic toxin is more frequent in atrophic rhinitis of swine. This gene was less frequent also in sheep and poultry strains [13]. Even in porcine toxigenic isolates, the presence of the gene proved to be low, and the gene is usually lost after a few subcultures. Some authors describe that the *toxA* gene encoding the toxin is not inserted into the bacterial chromosome but in a lysogenic bacteriophage that infects the agent [17].

Gene *pfhA* was also not found in any strain analyzed, which contrasts with that described by Ewers et al. [13], who found the gene in 25% (2/8) of isolates from dogs, 18.5% from cats (10/54), and 21.2% (11/52) from swine. Tang et al. [9] working with swine strains describe 15% of *P. multocida* tested positive to the *pfhA* gene (35/233). To date, no author describes the presence of this gene in *P. multocida* strains from rabbits.

The high prevalence of the *ptfA* gene (type 4 fimbriae) found in this study (93.4%-43/46) was expected since it is supposed to be a key element in fixing bacteria on the surface of the epithelial cells of hosts being rather common in rabbits [13].

The gene *tadD* has been described as a putative nonspecific tight adherence protein D in *P. multocida *[18] and was present in 91.3% (42/46) of rabbit strains. In swine strains this gene was described in 43.3% (100/233) of the isolates tested [9].

According to Atashpaz et al. [19] and Ewers et al. [13], the *tbpA* gene is closely related to ruminant strains (cattle, sheep, and buffalo), which explains their low frequency in the present study (8.6%-4/46).

Genes encoding proteins with different functions, such as iron acquisition (*exbBD-tonB, fur*), hyaluronidase (*pmHAS*), hemoglobin-binding proteins (*hgbA and hgbB)*, superoxidee dismutase (*SodA*, *Sod*C), porin *(psl*), neuraminidase (*nanH and nanB*), adesins (*fimA, hsf-1, hsf-2*), and membrane proteins (*oma87, ompH, plpB*), showed frequencies similar to those reported by Ewers et al. [13] and Tang et al. [9].

In contrast to what described by Tang et al. [9], the present study did not permit the association of a particular virulence factor to a specific capsular type, since the distribution of virulence factors was very heterogeneous among capsular type A and untypeable strains. It was observed that frequency of untypeable strains was significantly higher in strains isolated from herds B, C, and D, when compared to herd A.

The virulence genes profile generated were compared, and according to the presence or absence of tested genes the strains were clustered in 38 gene combinations with a high discrimination index (0.99). This information could not be compared to those found in the literature, but a strong association among strains from rabbitry and the animal were observed in clusters formed considering more than 80% of similarity. The virulence gene profile showed to be a good tool for the discrimination of *P. multocida* strains.

Infections caused by *P. multocida* are usually treated with a broad spectrum of antibiotics [20]. The antimicrobial susceptibility data from this study indicate that cephalosporins, florfenicol, tetracyclines, and fluoroquinolones are the most effective drugs, a fact also reported in studies conducted in France, North America, and Japan [20–22]. The high resistance of the isolates against sulfonamides and cotrimoxazole has also been previously described [9].

Rabbits have a growing role as companion animals and are traditionally used as a source of animal protein. The present study showed a relatively high frequency of agent isolation in Brazilian rabbits and a great potential of virulence and zoonotic transmission concerning the virulence gene profiles detected.

## Figures and Tables

**Table 1:** Frequency of positive animals in four rabbitries examined in São Paulo State, Brazil.

Year	Rabbitries	Number	Positive	Frequency (%)
2010	A	40	21	52.5
	B	20	2	10
2011	C	53	4	7.5
	D	27	2	7.4

Total		140	29	20.7

**Table 2:** Frequency of protein-coding genes and virulence factors in *P. multocida* strains isolated from rabbits.

Gene	Virulence factor	No. of positives/(%)
*toxA*	Dermonecrotic toxin	0/(0)
*pfhA*	Filamentous hemagglutinin	0/(0)
*hgbA*	Hemoglobin-binding protein	34/(73.9)
*hgbB*	Hemoglobin-binding protein	14/(30.4)
*exbBD-tonB*	Iron acquisition	28/(60.8)
*nanH*	Neuraminidase	31/(67.3)
*psl*	Porin	44/(95.6)
*nanB*	Neuraminidase	44/(95.6)
*ompH*	Outer membrane protein H	35/(76)
*oma87*	Outer membrane protein 87	39/(84.7)
*ptfA*	Type 4 fimbriae	43/(93.4)
*sodA*	Superoxide dismutase	35/(76)
*sodC*	Superoxide dismutase	45/(97.8)
*tbpA*	Transferrin binding protein	4/(8.6)
*fimA*	Fimbriae	30/(65.2)
*hsf1*	Autotransporter adhesion	13/(28.2)
*hsf2*	Autotransporter adhesion	31/(67.3)
*tadD*	Putative nonspecific tight adherence protein D	42/(91.3)
*fur*	Ferric uptake regulation protein	02/(4.3)
*pmHAS*	Hyaluronan synthase	35/(76.0)
*ompA*	Outer membrane protein A	13/(28.2)
*plpB*	Lipoprotein B	16/(34.7)
